# Elevated serum alpha-fetoprotein in poorly differentiated adenocarcinoma with neuroendocrine differentiation of the ascending colon: a case report

**DOI:** 10.1186/s12957-016-0838-0

**Published:** 2016-03-15

**Authors:** Hung-Hsin Lin, Chia-Chu Chang, Shung-Haur Yang, Shih-Ching Chang, Wei-Shone Chen, Wen-Yih Liang, Jen-Kou Lin, Jeng-Kai Jiang

**Affiliations:** Division of Colon and Rectal Surgery, Department of Surgery, Taipei Veterans General Hospital, No. 201, Sec. 2, Shih-Pai Rd., Taipei City, 11217 Taiwan; Institute of Clinical Medicine, National Yang-Ming University, No. 201, Sec. 2, Shih-Pai Rd., Taipei City, 11221 Taiwan; Department of Anesthesiology, Taipei Municipal Wan-Fang Hospital, Taipei Medical University, No. 111, Sec. 3, Xinglong Rd., Taipei City, 11696 Taiwan; Department of Pathology, Taipei Veterans General Hospital, No. 201, Sec. 2, Shih-Pai Rd., Taipei City, 11217 Taiwan

**Keywords:** Colon cancer, Poorly differentiated, Adenocarcinoma, Neuroendocrine, Alpha-fetoprotein

## Abstract

**Background:**

Colorectal cancer (CRC) is the most common form of cancer and the third leading cause of death in Taiwan. Serum alpha-fetoprotein (AFP) has been extensively used as a biomarker for hepatocellular carcinoma (HCC) and yolk sac tumors.

**Case presentation:**

This case report presents a 90-year-old woman with right abdominal pain and poor appetite for 1 week. The computed tomography (CT) showed wall thickening in the proximal ascending colon with ruptured appendicitis. Preoperative serum AFP was high. There was no definite liver metastasis or other abnormal findings in the hepatobiliary systems. After initial empirical antibiotic treatment, we performed laparoscopic right hemicolectomy. The pathological assessment was poorly differentiated adenocarcinoma with neuroendocrine differentiation in the ascending colon. The tumor cells did not produce AFP. Amazingly, the follow-up serum AFP level 1 month after the surgery declined to normal range. The patient had an uneventful course after the surgery and was free of recurrence or metastasis within 5 months of follow-up.

**Conclusions:**

AFP may be a useful tumor marker in poorly differentiated colorectal cancer with neuroendocrine component patients and a prediction of early treatment response.

## Background

Colorectal cancer (CRC) is the most common form of cancer and the third leading cause of death in Taiwan. Currently, more than 14,000 new cases of colorectal cancer are diagnosed annually [1]. Serum alpha-fetoprotein (AFP) has been extensively used as a biomarker for hepatocellular carcinoma (HCC) and yolk sac tumors [2, 3]. Elevated serum levels of AFP were also found in patients with carcinoma metastasis to the liver or non-neoplastic liver injury. Some studies have showed that the other tumors in human could also produce AFP, and gastric cancer was one of the most common [4]. Here, we report a rare case of colon cancer in a patient with an elevated serum AFP level. To the best of our knowledge, an elevated serum level of AFP resulting from colon adenocarcinoma with neuroendocrine differentiation has not been reported previously.

## Case presentation

A 90-year-old woman presented to the emergency department at the local hospital having had an intermittent abdominal pain with poor appetite for 1 week. Family history for colorectal cancer was negative.

**Fig. 1:**
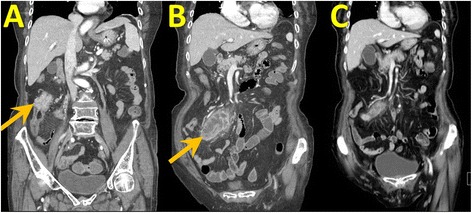
CT scans of the abdomen. An enhanced abdominal CT scan indicated wall thickening of the ascending colon, compatible with colon cancer (**a**, *arrow*), a swelling appendix with localized abscess, compatible with ruptured appendicitis (**b**, *arrow*), and regression of appendicular abscess after antibiotics treatment (**c**)

Preoperative imaging by abdominal computed tomography (CT) at the local hospital showed an annular tumor of 5.0 × 4.5 cm size in the ascending colon and wall thickening of the appendix, with peripheral fat stranding (Fig. 1a, b). Ascending colon tumor with ruptured appendicitis was diagnosed. There were no hints for other distant metastases, as revealed by CT and chest X-ray. Laboratory studies showed a hemoglobin concentration of 6.8 g/dL, WBC count of 15,300/μL, platelet count of 412,000/μL, and C-reactive protein level of 18.88 mg/dL. Standard serum tumor marker carcinogenic antigen (CEA) and CA19-9 were found normal, but AFP was elevated (90.1 ng/mL) according to a cutoff of 20 ng/mL. The patient had received 5-day intravenous empirical antibiotics in the local hospital before admission.

**Fig. 2:**
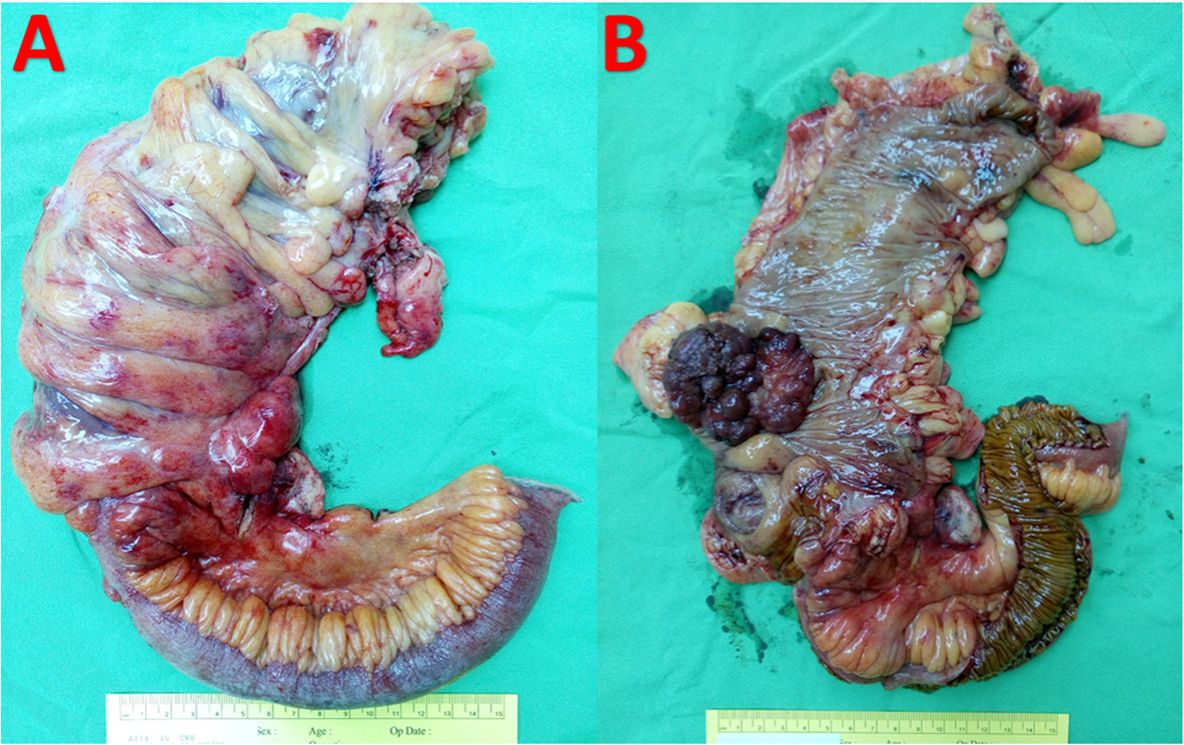
Resected ascending tumor. Gross appearance of the ascending colon tumor and ruptured appendicitis (**a**, **b**)

After admission to our hospital, a series of survey was performed, including abdominal CT (Fig. 1c). The general examination was unremarkable, but localized right lower quadrant abdominal tenderness, while laboratory studies showed a hemoglobin concentration of 9.8 g/dL, WBC count of 8000/μL, platelet count of 339,000/μL, and C-reactive protein level of 2.76 mg/dL. Renal and liver function tests were normal, and hepatitis B and C markers were negative. Serum carcinoembryonic antigen (CEA) and CA 19-9 levels were normal. However, AFP was still elevated (64.9 ng/mL). After completing preoperative diagnostics, emergent laparoscopic surgery was performed showing a fungating tumor involving the proximal ascending colon and a localized abscess between the terminal ileum and cecum, compatible with ruptured appendicitis (Fig. 2a, b). So, we performed laparoscopic right hemicolectomy with side-to-side anastomosis.

The pathological assessment of the resected specimen showed a 5 × 4 cm fungating tumor consisting of a poorly differentiated adenocarcinoma with neuroendocrine differentiation extending through the visceral peritoneum. Extramural vascular invasion of the adenocarcinoma cells was present. Microscopic evaluation of the 20 regional lymph nodes in the mesentery of the resected colon revealed four lymph nodes involvement. By immunohistochemistry, the cells were positive stained for CD20, CDX-2, synaptophysin, and negative for AFP, CD7, CD56, neuron-specific enolase, and chromogranin A. Morphologic and immunohistochemical findings confirmed the diagnosis of poorly differentiated adenocarcinoma with neuroendocrine differentiation within the ascending colon tumor and mesenteric lymph nodes. K-RAS and B-RAF genetic mutations were determined for primary tumor DNA after microdissection, and both genes were wild type. In summary, histological and immunohistochemical specifications finally revealed diagnosis of a poorly differentiated adenocarcinoma with neuroendocrine differentiation in the ascending colon and ruptured appendicitis. The final tumor stage was pT4aN2aM0, stage IIIc.

In the postoperative course with the ordinary ward, recovery with oral feeding starting at the fourth postoperative day was uneventful, the patient could be sent home 11 days after surgery. After discussion of this case in a multidisciplinary team treatment combined conference for colorectal cancer, the patient decided not to receive adjuvant chemotherapy because she was too weak to tolerate adjuvant chemotherapy. Interestingly, serum AFP level 1 month after the surgery declined to 1.99 ng/mL, used as tumor marker for recurrence. Other tumor markers (such as CEA and CA19-9) remained within the normal range during follow-up. Subsequent clinical and imaging follow-up revealed no signs of tumor recurrence up to now.

### Discussion

To our knowledge, this report is the first case of a poorly differentiated adenocarcinoma with neuroendocrine differentiation in the colonic segment, combined with an elevated serum AFP level. Surgery is the only treatment that has the potential to cure patients with CRC. In our case, there were no histopathological features and patterns of immunoreactivity typically usually seen in hepatoid adenocarcinoma.

In Taiwan, there is a large population of patients with hepatitis B or C virus infection. AFP is a tumor marker for HCC and had been used in the clinical practice for a long time. The high levels of AFP in this patient originally raised the question as to whether there was a tumor or another condition causing the expression of AFP. In our case, we performed CT image twice with no definite liver metastasis or hepatobiliary lesions.

To date, there is only limited data on most tumor markers measured in neuroendocrine tumor (NET) patients, which has thus created uncertainty about their role. According to the WHO classification, adenocarcinoma with neuroendocrine differentiation is distinguished from neuroendocrine carcinoma by <30 % of the neuroendocrine component [5]. Although overall AFP is elevated only in a minority of NET patient, this data analysis demonstrates the ability of AFP to highlight a group of NET patients with aggressive, high-grade tumors, and poor prognosis. Thus, AFP is likely to be a marker of tumor cell de-differentiation rather than a marker of hepatic metastases from NETs [3].

This was important for diagnosis of hepatoid adenocarcinoma, including immunoreactive AFP in the cytoplasm of cells in the trabecular or solid nests. In the aspect of growth pattern, poorly differentiated adenocarcinoma of solid type, small cell neuroendocrine cell carcinoma was similar to hepatoid adenocarcinoma. However, these tumors did not contain any cells positive for AFP, as our present case. Therefore, the diagnosis of hepatoid adenocarcinoma should be strictly based on combination of hepatoid features and AFP secretion.

In SEER database, the prognosis of adenocarcinoma with neuroendocrine differentiation (>30 % adenocarcinoma and <30 % neuroendocrine component) was closer to neuroendocrine carcinoma than to non-neuroendocrine high-grade adenocarcinoma [6]. This subtype of CRC was thought as high recurrence rates, especially liver metastasis. Relatively poor survival in poorly differentiated adenocarcinoma with neuroendocrine differentiation warrants studies of adjuvant systemic therapy. In our case, serum AFP levels were used as an indicator of therapeutic effectiveness and a marker for monitoring early recurrence and metastasis. The serum AFP levels apparently correlated with the tumor burden, and increasing AFP levels might give a warning for timely interventions.

## Conclusions

In this study, we demonstrated a patient with poorly differentiated adenocarcinoma with neuroendocrine differentiation in the ascending colon, with an elevated serum AFP. After curative radical colectomy, AFP declined to normal range. AFP may be a useful tumor marker in poorly differentiated CRC with neuroendocrine component patients, for early detection of tumor recurrence.

## Consent

Written informed consent was obtained from the patient for publication of this case report and accompanying images.

## Abbreviations

AFP: alpha-fetoprotein
CEA: carcinogenic antigen
CRC: colorectal cancer
CT: computed tomography
HCC: hepatocellular carcinoma
NET: neuroendocrine tumor
